# L-Ornithine Derived Polyamines in Cystic Fibrosis Airways

**DOI:** 10.1371/journal.pone.0046618

**Published:** 2012-10-05

**Authors:** Hartmut Grasemann, Darakhshanda Shehnaz, Masahiro Enomoto, Michael Leadley, Jaques Belik, Felix Ratjen

**Affiliations:** 1 Division of Respiratory Medicine, Department of Pediatrics, The Hospital for Sick Children, University of Toronto, Toronto, Canada; 2 Program in Physiology and Experimental Medicine, SickKids Research Institute, The Hospital for Sick Children, University of Toronto, Toronto, Canada; Johns Hopkins School of Medicine, United States of America

## Abstract

Increased arginase activity contributes to airway nitric oxide (NO) deficiency in cystic fibrosis (CF). Whether down-stream products of arginase activity contribute to CF lung disease is currently unknown. The objective of this study was to test whether L-ornithine derived polyamines are present in CF airways and contribute to airway pathophysiology. Polyamine concentrations were measured in sputum of patients with CF and in healthy controls, using liquid chromatography-tandem mass spectrometry. The effect of spermine on airway smooth muscle mechanical properties was assessed in bronchial segments of murine airways, using a wire myograph. Sputum polyamine concentrations in stable CF patients were similar to healthy controls for putrescine and spermidine but significantly higher for spermine. Pulmonary exacerbations were associated with an increase in sputum and spermine levels. Treatment for pulmonary exacerbations resulted in decreases in arginase activity, L-ornithine and spermine concentrations in sputum. The changes in sputum spermine with treatment correlated significantly with changes in L-ornithine but not with sputum inflammatory markers. Incubation of mouse bronchi with spermine resulted in an increase in acetylcholine-induced force and significantly reduced nitric oxide-induced bronchial relaxation. The polyamine spermine is increased in CF airways. Spermine contributes to airways obstruction by reducing the NO-mediated smooth muscle relaxation.

## Introduction

**Figure 1:**
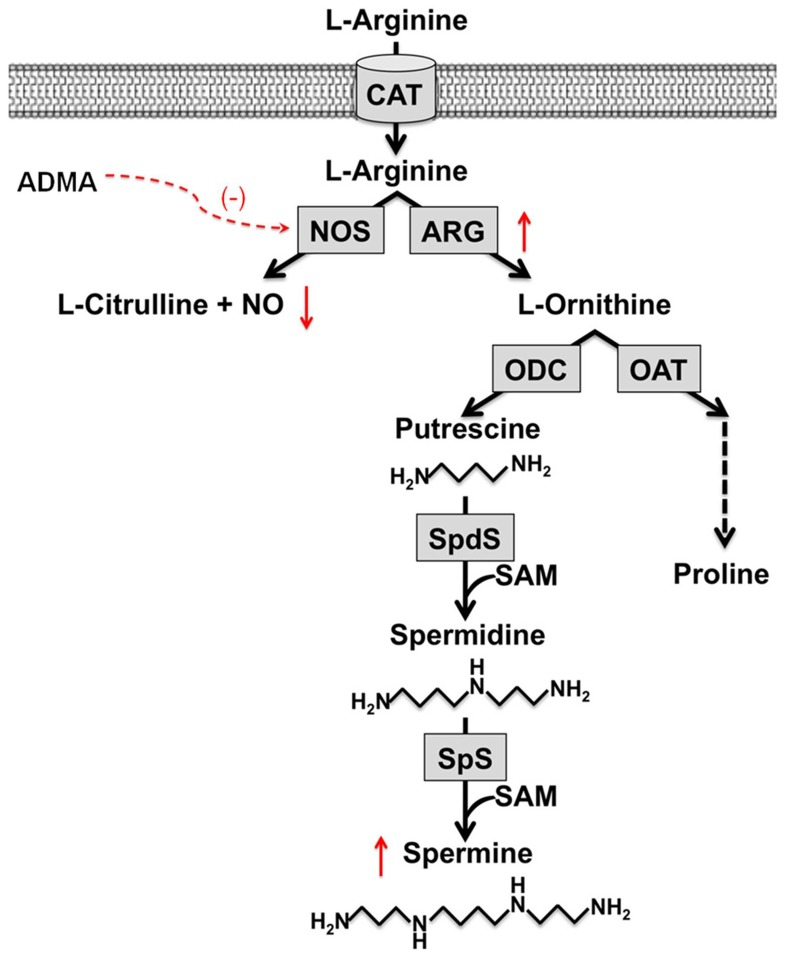
L-Arginine metabolism in cystic fibrosis sputum. ADMA: asymmetric dimethylarginine, ARG: arginase, CAT: cationic amino acid transporter, NO: nitric oxide, NOS: nitric oxide synthase, OAT: ornithine aminotransferase, ODC: L-ornithine decarboxylase, SAM: S-adenosylmethionine, SpS: spermine synthase, SpdS: spermidine synthase.

The amino acid L-arginine is substrate for enzymatic conversion by nitric oxide synthases (NOSs) and arginases. Recent evidence suggests an imbalance of the L-arginine metabolism in cystic fibrosis (CF) airways towards arginase as the activity of arginase is upregulated and levels of the endogenous nitric oxide synthase (NOS) inhibitor asymmetric dimethylarginine (ADMA) are increased in CF sputum. This imbalance contributes to the known decrease in CF airway nitric oxide (NO) production and results in increased concentrations of L-ornithine, the product of arginase activity [1]–[4] (Figure 1).

While it has been suggested that NO deficiency contributes to pathophysiology of CF lung disease and therapeutic interventions that improve airway NO production also result in improved pulmonary function in CF patients [5], little is known about the potential role of down-stream products of L-ornithine conversion. L-ornithine can be metabolized to proline, the precursor for collagen production by ornithine aminotransferase (OAT) and may as such contribute to pulmonary remodeling and fibrosis [6], [7]. L-ornithine is also substrate for ornithine decarboxylase (ODC), which catalyzes its conversion to putrescine, the first step in the polyamine biosynthesis. Putrescine is further metabolized to the higher-order polyamines, spermidine and spermine, by spermidine synthase and spermine synthase, respectively [7], [8] (Figure 1).

Polyamines are accumulated in alveolar airway epithelium by active transport, which may suggest that they could play an important functional role in airways [9], but their role in pulmonary diseases is currently unclear. Polyamines could contribute to lung damage, as back-conversion of the higher order polyamines results in formation of toxic compounds such as hydrogen peroxide [9]. Hydrogen peroxide, a product of and contributor to oxidative stress and cellular damage, may also induce contraction of airway smooth muscle [10]–[12]. On the other hand, polyamines could positively impact on bronchomotor tone, as they have been shown to relax guinea-pig tracheal smooth muscle [13]. Furthermore, polyamines may exhibit anti-inflammatory characteristics as spermine was shown to protect against lethal sepsis when administered systemically in mice [14].

While increased polyamine levels in blood and urine have previously been described in CF patients [15], [16] and urinary spermine excretion was positively correlated with CF lung disease severity [15], no information is available on polyamines in the airways of patients with CF. We therefore studied polyamines in CF by assessing polyamine profiles in sputum samples from stable CF patients and during periods of pulmonary exacerbation. In addition, we explored the functional role of the polyamine spermine on airway smooth muscle contraction and nitric oxide mediated relaxation. Some of the results related to this work have been previously reported in the form of an abstract [17].

## Methods

### Patients and study design

The study was approved by the Institutional Research Ethics Board; written informed consent was obtained in all cases. The diagnosis of CF had been confirmed by repeated sweat tests (chloride >60 mmol/L) and CFTR gene mutation analysis in all patients. Clinically stable patients were recruited during clinic visits. Sputum from CF patients with a pulmonary exacerbation was obtained within the first two days of hospital admission and after 14 days of intravenous (i.v.) antibiotic treatment. Healthy subjects had to be non-smokers and free of a respiratory tract infection for at least two weeks prior to recruitment.

Sputum from CF patients was collected after either spontaneous expectoration or induction with inhaled hypertonic saline as recently described [18]. Samples from patients with signs of pulmonary hemorrhage were excluded as the concentrations of spermidine and spermine were previously reported to be high [19]. Sputum production in all healthy controls was induced by hypertonic saline.

The non-liquid phase of sputum was processed by adding 0.1% DTT in D-PBS (4∶1 weight∶volume) and the resultant cell suspension was centrifuged to obtain cell-free clear supernatant, as previously reported [3]. The supernatant was aliquoted and stored at −80°C for subsequent analyses. Samples were deproteinized and cabamoylated. The carbamoylated polyamines were extracted with diethyl ether, separated by high-performance liquid chromatography (HPLC) and identified by liquid chromatography-tandem mass spectrometry (LC/MS/MS), as previously described [20]. Concentrations were determined by comparison to standard curves. L-ornithine was quantified using LC-tandem mass spectrometry [3], [21], sputum arginase activity and NO metabolite concentrations were measured, as previously described [1], [3].

### Organ bath studies

Airway smooth muscle mechanical properties were measured as previously described [22], [23]. Briefly, adult C57Bl/6 mice were sacrificed, 3^rd^–4^th^ generation intralobar bronchial segments from the left lung were mounted in a wire myograph and isometric changes were digitized and recorded (Myodaq, Danish Myo Technology A/S, Aarhus, Denmark). Tissues were bathed in Krebs-Henseleit buffer bubbled with air/6% CO_2_ and maintained at 37°C. Bronchial smooth muscle contractile response was normalized to the tissue cross sectional area (mN/mm^2^). The relaxant response to nitroprusside (SNP) was evaluated following stimulation with the E_75_ concentration of acetylcholine and expressed as a percentage of the contractile response in the absence and presence of spermine (10^−4^ M, 30 min pre-incubation). Procedures were conducted according to criteria established by the Canadian Council on Animal Care and were approved by the institutional Animal Care Review Committee.

### Statistical analyses

All results are expressed as the mean ± standard error of the mean (SEM). Binary comparisons were made with two-tailed student's t-test or Wilcoxon, where appropriate. Paired t-test was used to compare samples from CF patients before and after treatment for a pulmonary exacerbation. Correlations were determined using Spearman's test. P-values <0.05 were considered significant. Statistical analyses were conducted using GraphPad Prism 4.0c (Graphpad Software Inc., La Jolla, CA USA). The bronchial smooth muscle relaxant responses were evaluated by two-way ANOVA and multiple comparisons obtained by the Tukey-Krammer test.

## Results

The demographics of the study subjects are summarized in Table 1. A total of 10 healthy controls and 30 patients with CF (10 stable and 20 patients with a CF pulmonary exacerbation) were included. Sputum microbiology cultures in the stable patients showed no growth in 2 and were positive in 8 patients (*Pseudomonas aeruginosa* in 2, *Stenotrophomonas maltophilia* in 2, *Staphylococcus aureus* in 1, *Stenotrophomonas* and *Staphylococcus aureus* in 1, *Haemophilus influenza* and *Staphylococcus aureus* in 1, *Inquilinus limosus* in 1). Intravenous antibiotic treatments for exacerbations were given for 14 days and were directed against bacterial pathogens detected in patients' sputum culture which were *Staphylococcus aureus* in 12, *Haemophilus influenzae* in 3, and *Pseudomonas aeruginosa* in 5 patients.

**Table 1:** Demographics of study populations.

	N (gender)	Age	FEV_1_
**Healthy controls**	10 (6F/4M)	18.9 (14–22) yrs	
CF stable	10 (5F/5M)	13.0 (9–17) yrs	82.8 (60–102)%
CF exacerbation	20 (10F/10M)	12.6 (6–17) yrs	53.1(36–69)%

Gender: F = females, M = males. Age: numbers represent mean (range) age in years. FEV_1_: values represent FEV_1_ in % of predicted vales (24).

**Figure 2:**
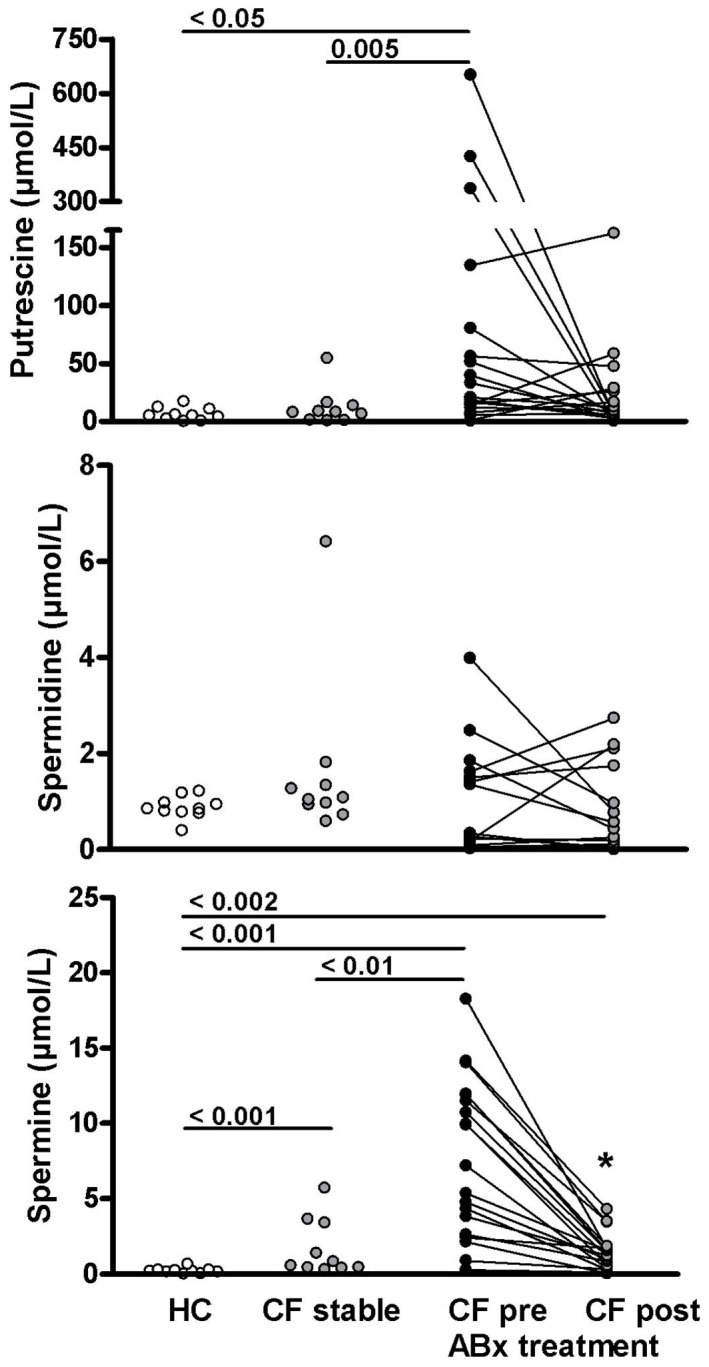
Sputum concentrations of putrescine, spermidine and spermine in healthy controls (HC), stable cystic fibrosis (CF), and CF patients with a pulmonary exacerbation before (pre) and after (post) treatment with intravenous antibiotics (ABx). Each dot represents an individual patient. Numbers indicate p-values for respective group comparison. * = p<0.0001 comparing pre vs. post treatment samples (paired t-test).

**Figure 3:**
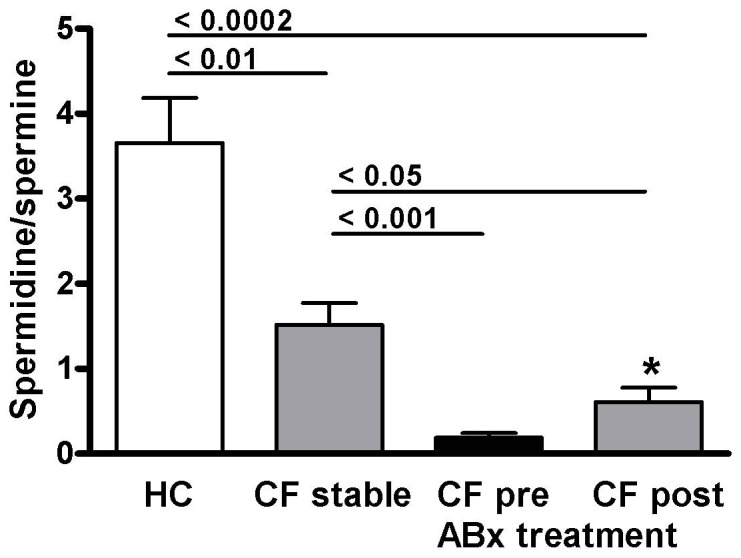
The ratio of spermidine/spermine in sputum of healthy controls (HC), stable cystic fibrosis (CF), and CF patients with a pulmonary exacerbation before (pre) and after (post) treatment with intravenous antibiotics (ABx). Results are expressed as means ± SEM. Numbers indicate p-values for respective group comparison. * = p<0.001 comparing pre vs. post treatment samples (paired t-test).

The ornithine-derived polyamines putrescine, spermidine and spermine could be measured in all sputum samples. Mean (± SEM) concentrations in healthy control samples were 11.91±5.03 µmol/L for putrescine, 0.88±0.07 µmol/L for spermidine and 0.22±0.06 µmol/L for spermine (Table 2). Sputum polyamine concentrations in stable CF patients were similar to healthy controls for putrescine (6.18±1.73 µmol/L) and spermidine (1.62±0.54 µmol/L) but were significantly higher for spermine (1.71±0.60 µmol/L, p = 0.0002) (Figure 2). Spermine concentrations in the two stable CF patients with sterile sputum samples were 0.55 and 0.45 µmol/L, respectively. The spermidine/spermine ratio, which was previously reported to be increased in serum and urine of CF patients [18], was significantly reduced in stable CF sputum compared to healthy controls (1.51±0.26 vs. 3.66±0.53, p<0.01) (Figure 3). In stable CF sputum there was a positive correlation of L-ornithine with both spermidine (R = 0.817, P = 0.01) and spermine (R = 0.838, P<0.005) but not with putrescine concentrations. There was no correlation of sputum polyamine levels and age.

**Table 2:** L-ornithine derived polyamine concentrations in sputum.

	Putrescine	Spermidine	Spermine	Spd/spm
**Healthy controls**	11.91±5.03	0.88±0.07	0.22±0.06	3.66±0.53
**CF stable**	6.18±1.73	1.62±0.54	1.71±0.60	1.51±0.26
**CF exacerbation**				
**pre**	96.02±38.65	0.78±0.24	7.32±1.19	0.19±0.05
**post**	20.59±8.25	0.62±0.19	1.35±0.27	0.61±0.17

Data shown are mean ± SEM concentrations in µmol/L. For cystic fibrosis (CF) patients with an exacerbation sputum concentrations are shown before (pre) and after (post) antibiotic treatment.

The concentrations of L-ornithine derived polyamines were highest in CF patients presenting with a pulmonary exacerbation. In comparison to stable CF, patients with pulmonary exacerbation had higher concentrations of putrescine (96.02±38.65 µmol/L, p = 0.005), and spermine (7.32±1.19 µmol/L, p = 0.006). In contrast, mean spermidine concentrations in samples from patients with an exacerbation tended to be lower than in stable CF, although this difference did not reach statistical significance (0.78±0.24 µmol/L, p = 0.075) (Figure 2). The spermidine/spermine ratio was 0.19±0.05 in sputum from patients with an exacerbation, which was significantly lower than in stable CF or healthy controls (<0.0001, respectively) (Figure 3).

Treatment for pulmonary exacerbations resulted in a non-significant decrease in sputum putrescine to 20.59±8.25 µmol/L (p = 0.075), and a significant decrease in spermine to 1.35±0.27 µmol/L (p<0.0001). There was no change in spermidine concentration with antibiotic treatment (0.62±0.19 µmol/L) (Figure 2). The spermidine/spermine ratio significantly increased to 0.61±0.17 (p = 0.0007) (Figure 3). Spermidine concentrations after antibiotic treatment were lower than in stable CF (p = 0.0089) and healthy controls (p = 0.033). Spermine concentrations after treatment were not different from stable CF but remained increased compared to healthy controls (p = 0.0017). Despite the increase in spermidine/spermine with treatment, the ratio remains significantly lower compared to both stable CF (p = 0.02) and healthy controls (p = 0.0002) (Table 2) (Figure 3).

**Figure 4:**
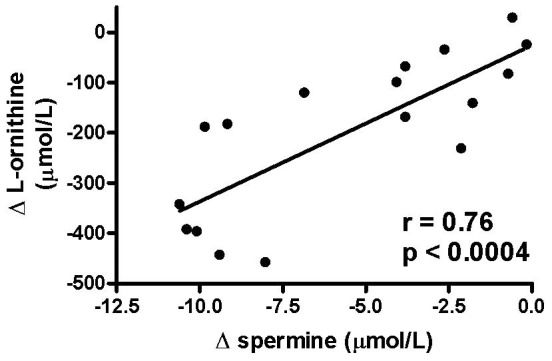
Correlation of changes (Δ) in sputum L-ornithine and spermine concentrations in cystic fibrosis patients treated for a pulmonary exacerbation . Each symbol represents one individual.

As expected, treatment for pulmonary exacerbations resulted in a significant improvement in FEV_1_ of 13.4 (−8 to 30)% predicted to an FEV_1_ of 66.5 (45–93)% of predicted after treatment. This was associated with a decrease in sputum arginase activity (276.7±39.4 vs. 115.8±33.3 mUnit/mg protein, p = 0.0023) and in the concentration of ornithine, the product of arginase activity (275.5±46.85 vs. 78.01±18.3 µmol/L, p = 0.0005), while NO-metabolite levels significantly increased (477.3±93.2 vs. 835.1±127.5 µmol/L, p<0.01). Antibiotic treatment also resulted in a decrease in sputum neutrophil counts (90.2±2.4 vs. 14.6±4.0×10^6^/g, p = 0.0001), neutrophile elastase activity (2.59±0.28 vs. 1.29±0.23 U/ml of sputum, p = 0.0005) and IL-8 concentration (178.8±19.9 sv. 87.02±18.2 ng/ml of sputum, p = 0.0026). The changes in either of the L-ornithine derived polyamine concentration with treatment for pulmonary exacerbations did not correlate with changes in FEV_1_, sputum NO-metabolites, neutrophil cell counts, neutrophil elastase activity, or IL-8 concentration in sputum. However, changes in sputum NO-metabolites correlated with changes in FEV_1_ % predicted (R = 0. 67, p = 0.004). There was also a significant correlation of the changes in spermine concentration and changes in sputum L-ornithine levels with treatment (spearman R = 0.76, p = 0.0004) (Figure 4).

**Figure 5:**
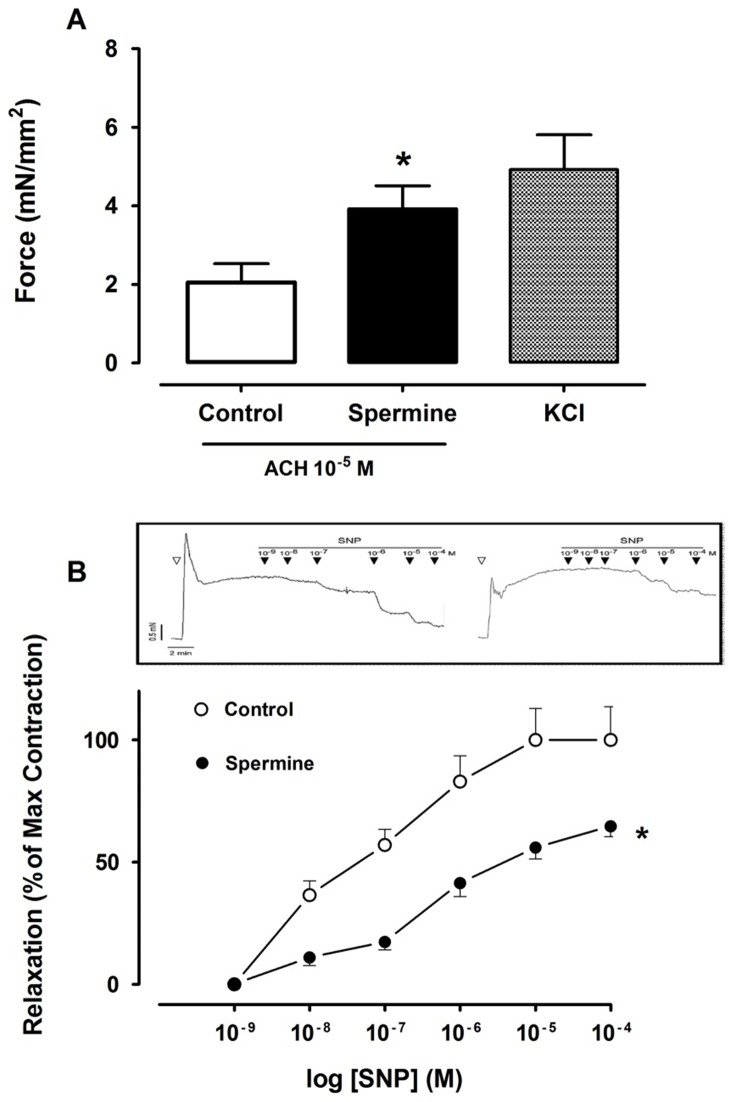
Spermine effect on mice bronchial smooth muscle contractile properties. A) Bronchial rings stimulated with acethylcholine (10−5 M) in the absence (Control; N = 6 rings) and presence (N = 10 rings) of spermine (10−4 M) or KCl (128 mM). B) Sodium nitroprusside (SNP) dose-response relaxation curves of acetylcholine pre-contracted (E75 = 10−5 M) bronchial smooth muscle in the absence (Control; N = 6 rings) and presence (N = 10 rings) of spermine (10−4 M). A typical SNP-induced relaxation tracing in the absence (left panel) and presence (right panel) of spermine is shown. Following ACH Stimulation (open triangle) the sustained bronchial muscle force in the presence of spermine is greater than in controls. * P<0.01 versus control by unpaired Student t-test (A) or two-way ANOVA with Tukey-Kramer multiple comparison testing (B).

The effect of spermine on bronchial smooth muscle was evaluated in vitro in bronchial segments obtained from adult mice. Incubation of the bronchi with spermine resulted in a near two-fold increase in acetylcholine-induced force (P<0.01), when compared to the untreated bronchial segments (Figure 5 A). Spermine also significantly reduced the SNP-induced bronchial muscle relaxation (P<0.01). When compared to bronchial segments, a 60% reduction in SNP-induced relaxation was documented in spermine-exposed bronchi at the maximum SNP concentration tested (Figure 5 B).

## Discussion

While previous studies looking at imbalances in the L-arginine metabolism in cystic fibrosis have focused on the role of changes in NO production on airway physiology [1]–[4], the effects of downstream products of increased arginase activity for airways function were largely unknown. We here studied the concentrations of the L-ornithine derived polyamines putrescine, spermidine and spermine in sputum of patients with CF and healthy controls. The main findings of our studies are that (i) in clinically stable CF patients sputum levels of putrescine and spermidine were similar to those found in healthy controls, while concentrations of the higher order polyamine spermine were significantly increased. (ii) In patients presenting with a CF pulmonary exacerbation the sputum levels of spermine were highest and treatment resulted in a decrease in spermine to values similar to stable CF patients. (iii) Experiments in isolated mouse airways showed that the addition of spermine resulted in an enhanced contractile response to muscarinic agonist stimulation, but reduced relaxation to the NO-donor SNP.

Elevated polyamine levels have previously been described in CF patients for blood and urine [15], [16]. Of interest, an increased ratio of spermidine/spermine was found in whole blood and red blood cells of CF patients compared to controls [19]. This was in contrast to our findings in sputum where the spermidine/spermine ratio was lower than normal in stable CF and even further decreased in CF patients with an exacerbation. The decrease in the ratio can be explained by the fact that there was a significant increase in sputum spermine but no difference in spermidine when comparing CF with controls in CF. As polyamines are synthesized and metabolized in many organisms including bacteria it is conceivable that organisms colonizing the CF airways also contribute to and/or metabolize sputum polyamines. However, the increase in spermine in CF sputum cannot be explained by the presence of bacteria alone, as the sterile sputum samples obtained from two stable CF patients showed approximately 2-fold higher concentrations than the average found in healthy controls. The observation that spermine concentrations were highest in CF patients presenting with a pulmonary exacerbation and decreased with antibiotic treatment may however suggest that bacteria could be a source of increased sputum polyamine concentrations during a CF pulmonary exacerbation. Alternative explanation for increased spermine in CF sputum includes accumulation due to reduced back-conversion of spermine [24], [25].

The decrease in spermine concentrations during treatment correlated significantly with the decrease in sputum L-ornithine, the product of arginase activity. This pattern is consistent with the observed changes in arginase activity with treatment for a CF pulmonary exacerbation in this and previous studies [1]–[3] and both production of L-ornithine by arginase and conversion of L-ornithine to putrescine by ODC can be rate-limiting steps in the polyamine synthesis [26]. However, the expression or activity of ODC in CF airways was not assessed in our study.

The functional studies in isolated mouse airways demonstrate that an increase in spermine in the airways may contribute to a higher airway resistance by reducing the epithelial-dependent dilatatory effect of NO. Also, spermine acts as competitive inhibitor of NOS in various cell types, although it remains unclear whether inhibition of NOS occurs in the lung at physiological polyamines concentrations. The inhibitory effect of spermine on NOS1 (neuronal NOS), the NOS isoforms most critical for the regulation of broncho-motor tone [27], was found to have an IC_50_ value of 56 µM [28]. The measured concentrations of spermine in sputum from CF patients with exacerbation were also in the µmolar range with a mean of 7.3 µM and a max of 18.25 µM; however tissue concentrations of spermine may be even higher than in sputum as polyamines may accumulated in lung tissue by active transport, as demonstrated for alveolar epithelial cells [9]. The IC_50_ of spermine for NOS2 (inducible NOS), an enzyme that has been shown to be expressed in CF airway epithelial cells at significantly reduced levels [29], was reported to be higher than for NOS1, i.e. 500 µM (using lysates from endotoxemic rat liver) [30]. Therefore, at the concentrations measured in the CF sputum samples, spermine is more likely to effectively inhibit NOS1 than to have an inhibitory effect on NOS2.

Although there appeared to be no direct correlation of spermine with sputum NO metabolite concentrations or with pulmonary function, changes in sputum NO-metabolites during treatment for a CF exacerbation correlated significantly with changes in FEV_1_ % predicted, which was consistent with previous observations from our group [3]. Relevant to this finding and of possible importance to the mechanism accounting for the spermine effect on NO-mediated airway tone is the observation that spermine can inhibit L-arginine uptake through the cationic amino acid transporter CAT-2, and thereby reducing L-arginine availability for intracellular NOS and NO production, as recently demonstrated in macrophages [31]. Reduced L-arginine availability is thought to contribute to the known NO deficiency and to airway obstruction in CF patients [1]–[3]. Recent evidence suggests that airway smooth muscle cell contraction may also be controlled by CAT-2 through a spermine-dependent pathway [32].

Interestingly, the experiments with precontracted isolated airway rings demonstrated that spermine prevented the expected relaxation by the NO-donor SNP. While the underlying mechanism for this effect is unclear, the observation suggests that spermine may indirectly contribute to airway constriction by reducing the availability of NO for bronchodilation. As spermine is known to bind free radicals [33], one possible explanation for our observation is that spermine may also act as NO scavenger. Alternatively, spermine may exert a direct effect on airway constriction as suggested by the increased force generated by airway smooth muscle incubated with spermine; experiments that were conducted in the absence of a NO donor. Contractile effects of spermidine and spermine on airways were previously demonstrated in epithelial-denuded rat tracheal segments [33], [34]. In contrast, spermidine and spermine induced transient guinea-pig trachea relaxation [13], while increased Ca^+2^-activated force generation and shortening velocity was reported in guinea pig ileum in the presence of spermine [35]. Besides effects on airway tone, an increase of polyamines could also have other potential consequences for CF lung disease. For instance, polyamines may exhibit anti-inflammatory characteristics as spermine was shown to protect against lethal sepsis when administered systemically in mice [14]. We did not find any evidence for an anti-inflammatory effect of polyamines in the CF airways, based on the absence of correlations between polyamine concentrations or the changes in spermine during treatment with established sputum markers of airways inflammation (i.e. neutrophil counts, neutrophil elastase activity, or IL-8 levels), or with NO metabolite concentrations.

In summary, we found increased concentrations of the L-ornithine derived polyamine spermine in the airways of patients with CF. Ex-vivo data from the mouse suggested that spermine may influence bronchomotor tone directly and by inhibiting NO-mediated bronchodilation. Further studies are needed to evaluate the potential effects of increased spermine concentrations on other aspects of CF lung disease such as bacterial infection and airway inflammation.
